# Community Dynamics of Carrion Flies and their Parasitoids in Experimental Carcasses in Central Argentina

**DOI:** 10.1673/031.012.0801

**Published:** 2012-01-25

**Authors:** Moira Battán Horenstein, Adriana Salvo

**Affiliations:** ^1^ Cátedra Diversidad Animal I, CONICET; ^2^ Centro de Investigaciones Entomolóacute;gicas de Córdoba, IMBIV/CONICET; ^3^ Facultad de Ciencias Exactas Físicas y Naturales, Universidad Nacional de Córdoba, Av. Velez Sarsfiel 299, Córdoba, Argentina

## Abstract

Insects are the predominant group regarding both species richness and abundance that develop on carrion. Among them, the most important decomposers using carrion as a source of food for their development are the immature stages of the dipteran families Calliphoridae, Muscidae, and Sarcophagidae. The large numbers of their larvae in carcasses are attacked by a rich community of parasitoids, including species of Braconidae, Ichneumonidae, and Pteromalidae (Hymenoptera: Parasitica). The objective of this study was describing the temporal composition and dynamics of a parasitoid community in relation to their dipterans hosts in carrion in terms of number of species and specific composition, irrespective of the particular interactions between species in both trophic levels. Additionally, seasonality of the climate in the region was investigated as a factor structuring the studied communities. The experiments were undertaken in the south of Cordoba, Argentina during 2004 in a rural area. Two traps per season were placed separately approximately 300 m from each other in the study site. Each trap contained a domestic pig (*Sus scrofa*) of approximately 8 kg as bait. Samples were taken daily during the first four weeks and then every two or three days over the following weeks until the end of the experiment. The dipteran community was represented by 15 species in 6 families of the Calyptratae Diptera whereas parasitoids belonged to six families of the parasitic Apocrita Hymenoptera. Climatic seasonality was an important factor in determining the number of occurring species in the carcasses and community composition. The highest number of species was observed in the spring for both communities.

## Introduction

Information concerning dynamic of host— parasitoid communities is valuable from several points of view. First of all, parasitoid wasps and their insect hosts comprise about one—third of all animal species, and more than 50% of all terrestrial animal species; thus, understanding the way in which their communities are structured represents an relevant topic in ecology (Bailey et al 2009). In the other hand, parasitoids act as important regulators of their insect host populations in both natural and managed habitats (LaSalle 1993), and have been largely employed as biological control of insect pests. This fact is relevant because Dipteran species are important in the degradation of organic matter, and also have medical and veterinary importance as they can be mechanical vectors of biological pathogens, and some species cause myiasis to humans and other vertebrates (Greenberg 1971; Ferreira 1978; Linhares 1981; Guimaraes et al. 1983). For this reason, population dynamics and community organization of host—parasitoid systems have been extensively studied in the context of crop pest management, and very infrequently in other type of systems involving carrion or dung (Marchiori and Linhares 1999; Marchiori et al. 2000; Castillo 2001).

Carrion and other kinds of decaying organic matter like fungi, dung, or fruit are nutritionally rich but discrete and ephemeral resources in which insects are the predominant group in both species richness and abundance (Payne 1965; Hanski 1991). Among the necrophagous species, the dipteran families Calliphoridae, Muscidae, Sarcophagidae, and Fanniidae (frequently named as “carrion flies”) are considered the most important decomposers because their immature stages use carrion as a source of food for their development (D'Almeida 1993). In this type of system, the large number of larvae of carrion flies feeding on organic matter and their pupae attract a rich community of parasitoids including species of Braconidae, Ichneumonidae, Pteromalidae, Figitidae, Eulophidae, Chalcididae, and Diapriidae families (Hymenoptera Parasitica) (Figg et al. 1983; Cervenka and Moon 1991), and are expected to experience high levels of parasitism (Atkinson and Shorrocks 1981). Parasitism at immature stages has been mentioned as having an important influence in defining the community structure of blow flies (Beaver 1977).

Climate has a great influence on several aspects of insect communities. For example, it has been demonstrated that in temperate zones, climate is the most influential factor on Lepidopteran species richness through both direct effects (higher temperature may correlate with higher numbers of species) and indirect (weather influences on food availability) effects (Checa et al. 2009). Marked seasonality in the southern zone of the Neotropical region might have a direct influence on structuring communities of insects on carrion as a consequence of the dissimilar preferences or tolerance of the species in the communities by climatic conditions. Moreover, an indirect effect of weather may be evident: length of decomposition time of the carcasses is highly affected by climatic conditions (Campobasso et al. 2001). Thus, the different ability of the species to exploit these ephemeral resources may determine the structure of the community. Therefore, it should be important not only to describe patterns of species richness but also it variation throughout the year.

This study aimed to describe the temporal composition and temporal dynamics of the parasitoid community in relation to their dipterans hosts in carrion, in terms of number of species and specific composition irrespective to the particular interactions established between species in both trophic levels. Additionally, the marked seasonality of the climate in the region was investigated as a relevant factor in structuring the studied communities. A number of studies from temperate and tropical regions of the world have focused on the dipteran community using carrion as source of food and shelter from an ecological or forensic perspective (Braack 1987; Anderson and VanLaerhoven 1996; Souza and Linhares 1997; Centeno et al. 2002; Battán Horenstein et al. 2007, 2010), but very few included the upper trophic level (parasitoids) associated with these communities (Castillo 2001). To the best of our knowledge, this is the first study that simultaneously examines the temporal community dynamic of both blow flies and their parasitoids in the southern Neotropical region.

## Materials and Methods

### Study site

The experiments were undertaken in the south of Cordoba, Argentina, an area characterized by the presence of algarrobo trees, namely *Prosopis alba* Grisebach (Favales: Fabaceae) and *P. nigra.* However, over the last 100 years this vegetation has been partially replaced by cultures of citrus, soybean, and alfalfa. The weather is predominantly dry and cold between March and September (autumn and winter), and warm and wet from September to March (spring and summer), with an annual rainfall ranging between 800–1000 mm (citations). The area can be defined as rural, as it has dense vegetation and isolated houses. The site chosen for experiments was a transitional area between natural and urban regions; thus, biodiversity was expected to be high because of the diversity in habitat types.

### Insect sampling

A variant of the trap designed by Schoenly et al. (1991) was used measuring 120 × 90 × 60 cm, which was designed to collect arthropods attracted to the carcass bait and those leaving it, including the successfuly reared offspring of those females which oviposit on the corpse. Two traps per season in 2004 were placed separately approximately 300 m from each other in the study site. Each trap contained a domestic pig (*Sus scrofa*) of approximately 8 kg as bait that had been killed by a sharp blow to the head with a blunt metallic object and immediately placed in the trap. Each experiment lasted until the entire carcass was consumed (approximately 7 weeks). Samples were taken daily during the first four weeks in order to collect dipteran and hymenopteran parasitoid species, and then every two or three days over the following weeks until the end of the experiment. Relative humidity and temperature in both the traps and in the vicinity of the traps were recorded daily with a portable thermo hygrometer (Hygro/In/Out Thermometer HT05). Meteorological data for each season were obtained from the local weather station, located approximately 15 km from the study site.

Variables obtained were the number of species, larvae, adult flies, and adult parasitoids. Analysis was restricted to parasitoid species mentioned as commonly associated to blow flies in the literature. It should be noted that most of the parasitoid species recorded in this study are gregarious (except *Alysia alticola* Ashmead (Hymeonoptera: Baraconidae) and *Brachymeria* podagrica Fabricious (Chalcididae)), thus any conclusion about their abundances based on rearing will be biased depending on the number of adults produced by hosts. However, our analyses based on parasitoid numbers do not refer to parasitism percentages, but the pool of parasitoids available to parasitize hosts in the next generation.

### Statistical analysis

Data obtained from the two placed traps on each season were pooled for statistical analysis. Species richness (average and cumulative) and indices of diversity (Shannon-Weaver) for dipteran and parasitoid communities were calculated by pooling data per season. Similarities in the taxonomic structure of communities along the seasons was studied via principal component analysis, using the standarized abundance of species as variables. Spearman correlation coefficient was used to analyze the relationship between climatic variables (precipitation, maximum and minimum temperature) and the abundances and community species richness of flies (larvae and adults) and parasitoids. Using the same coefficient, the correlation among abundance of larvae of dipteran hosts and the abundance and richness of associated parasitoids was investigated, partitioning data according seasons. All statistical analyses were performed using the Infostat version 1.1 statistical package.

**Figure 1.**
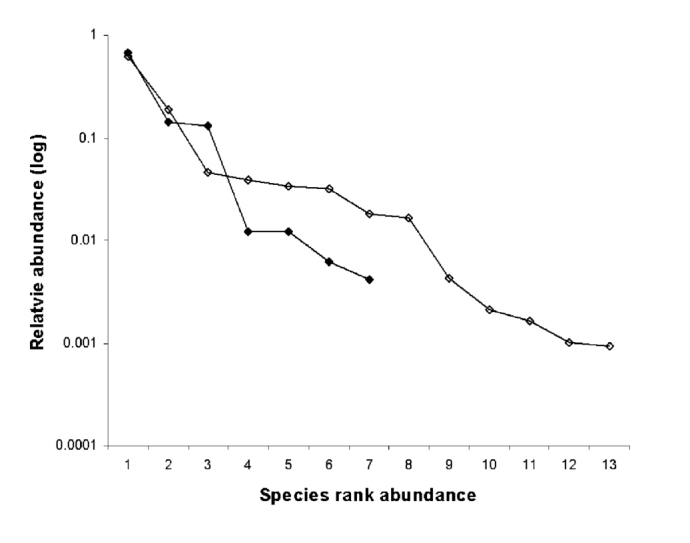
Species rank abundance of fly (empty circles) and parasitoids (black circles) communities. High quality figures are available online.

## Results

Accumulated data of trapped adults (N = 25,690) showed that the distribution of individuals into the species was very similar and fitted to log series models in both fly (χ^2^ = 13.43 df = 13) and parasitoid (χ^2^ = 9.82 df = 9) communites (Figure 1).

The dipteran community was representated by a total of 24,252 adult flies reared during the four experiments, which belonged to 15 species in six families (Table 1). Calliphoridae was the dominant family of the dipteran community collected (74% of reared adults), with Muscidae being the second most abundant family (more than 20%) except in winter, when Fanniidae became the second most abundant family (Table 1). *Chrysoma albiceps* Wiedemann (Calliphoridae) was the dominant species, accounting for more than 60% of total adults in the dipteran community.

**Figure 2.**
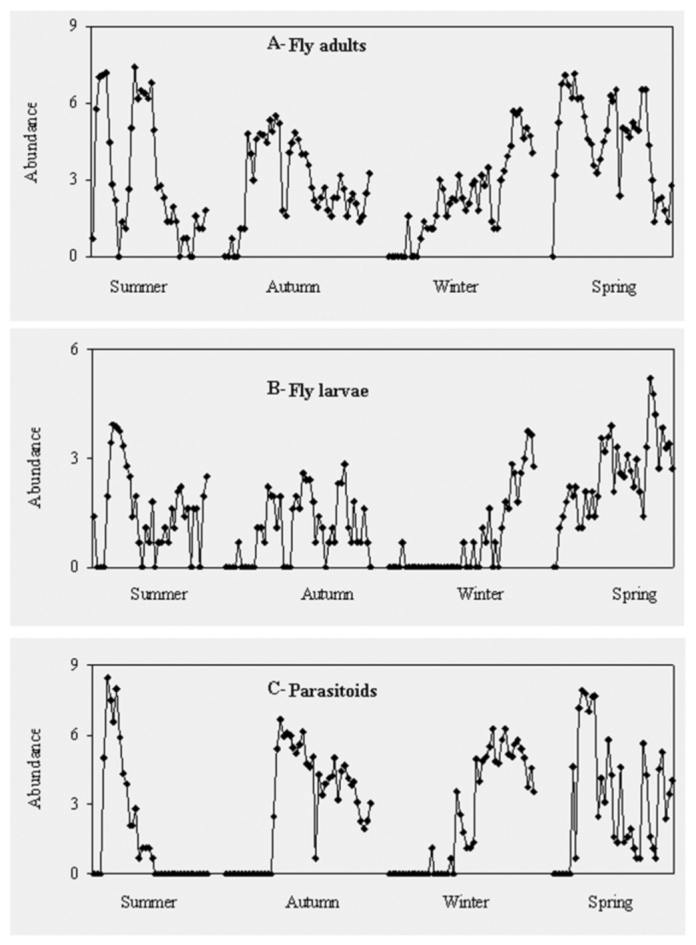
Fluctuations in abundance (log N+1) of fly adults (diamonds), fly larvae (empty diamonds), and parasitoids (squares). High quality figures are available online.

**Figure 3.**
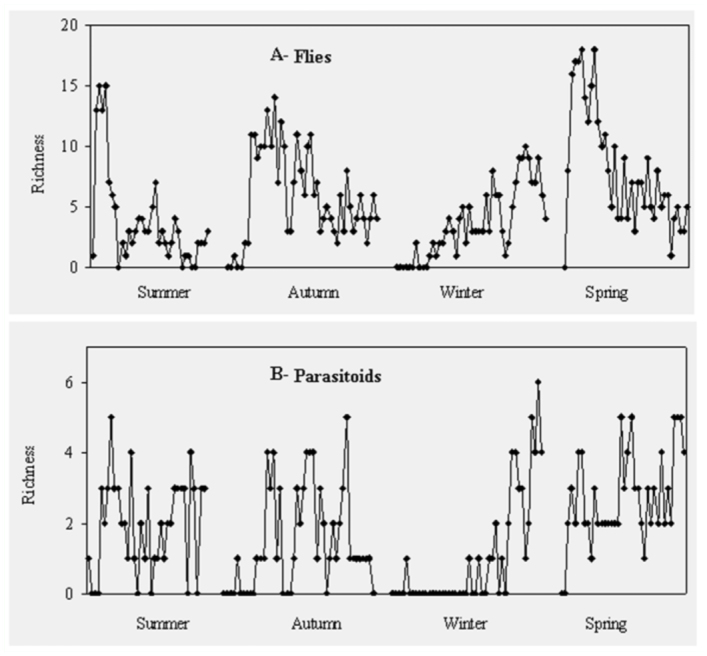
Fluctuations in richness of fly (diamonds) and parasitoid (squares) communities based on adult records. High quality figures are available online.

A total of 1438 adult parasitoids belonging seven species in six families of Hymenoptera were collected (Table 1). The pteromalid *Nasonia vitripennis* Ashmead (Pteromalidae) was the most abundant parasitoid species accounting for 69% of total obtained parasitoids. *Aphaereta* sp. (Braconidae) and *Tachinaephagus zaelandicus* Ashmead (Encyrtidae) were the second and third most abundant species in with 15 and 13% of the obtained adults respectively. Other species were represented by just 1% of reared adults.

The arrival of dipteran species each season was during the early days of decomposition of the carcasses, whereas parasitoid species arrived to carcasses a few days later, coinciding with the presence of abundant dipteran larvae (Figure 2). The two variables, dipteran and hymenopteran adults, were positively correlated in winter (r = 0.80, *p* < 0.01) and in autumn (r = 0.45, *p* < 0.01). Correlations were not significant in the other seasons. The peak of abundance of parasitoid species was closely related to the presence of abundant dipteran larvae, mainly in summer (r = 0.64, *p* < 0.01). Parasitoid abundance was also positively correlated to the richness of fly species in winter (r = 0.67, *p* < 0.01) and spring (r = 0.35*, p* < 0.05) (Figure 3), but non—significant correlations between variables were observed in summer and autumn.

**Figure 4.**
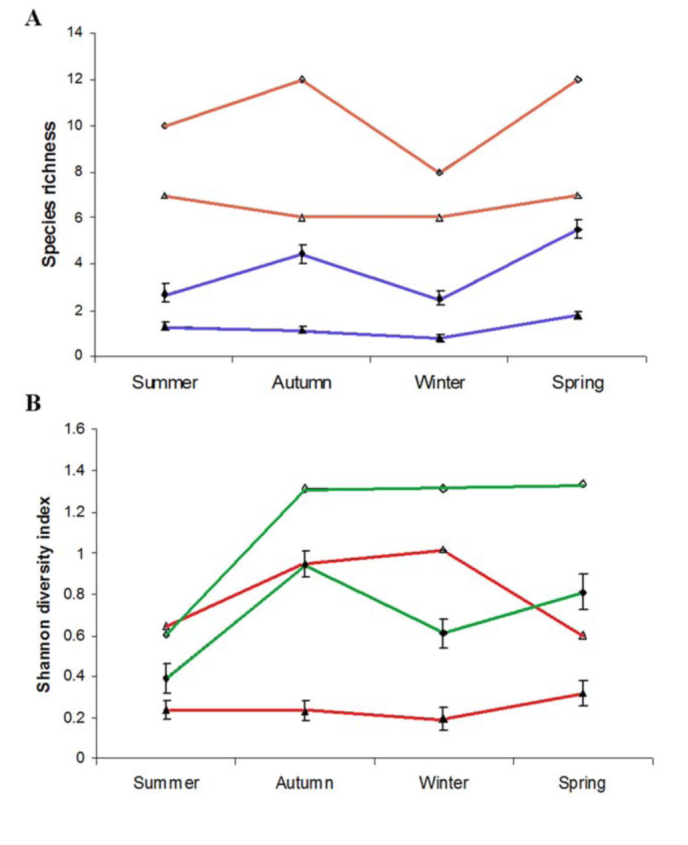
Averages (filled symbols) and values accumulated per season (empty simbols) of (A) species richness and (B) Shannon Diversity index of fly (diamonds) and parasitoid (squares) communities. Vertical bars are two standard errors, n = 36 in summer, 41 in autumn, 41 in winter, and 38 in spring. High quality figures are available online.

The dipteran community was richer and more diverse than the parasitoid community during all sampling periods and, in general, winter was the season with lower species richness and lower diversity indices in both fly and parasitoid communities (Figure 4). This trend was observed in the average and cumulative values of richness and diversity indices. Several positive and significant correlations between climatic variables and most community variables were observed (Table 2).

**Figure 5.**
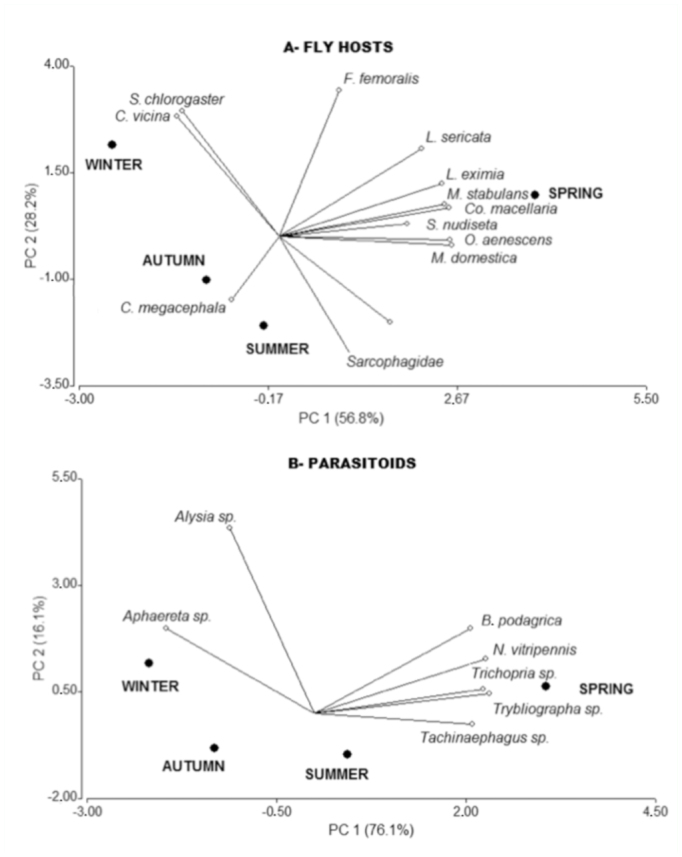
Principal component analysis of (A) blow flies and (B) their associated parasitoid communities in carcasses exposed in the four seasons. High quality figures are available online.

The occurrence and abundance of fly and parasitoid species notably varied with the season (Figure 5). In both fly and parasitoid communities, winter and spring were the most different seasons according the relative representation of species. In winter, just a few species prevailed: *Callipora vicina* Robineau-Desvoidy (Calliphoridae) and *Sarconesia chlorogaster* Wiedemann in the fly community, and *Aphaereta* sp. and *A. alticola* among the parasitoids. Spring samplings were characterized by the highest number of species in both fly and parasitoid communities (Figure 5).

## Discussion

Diptera and Hymenoptera communities in pig carcasses observed in this study were similar to those reported in other geographic regions (Payne 1965; Tantawi et al. 1996; Marchiori and Linhares 1999; Marchiori et al. 2000; Castillo 2001).

The abundance of the majority of the dipteran and hymenopteran species was positively correlated to temperature, and an increment in their numbers was related to the increment of precipitation.

Taxonomic structure of dipteran and parasitoid communities, as revealed by the PCA analysis, suffered a pronounced change across seasons. This could reflect interspecific variation in both competitive abilities and climatic preferences. Differences observed in the dipteran community were due to dominance of *C. albiceps* or *M. domestica* in spring (Battán Horenstein et al. 2007, 2010) and *S. chlorogaster* and *C. vicina* in the winter.

The most severe climatic conditions typical of winter and summer (very low and high temperatures, respectively) negatively impacted the dipteran community, which presented the lowest values for number of species and species diversity in these extreme seasons. The highest number of species and highest diversity indices were observed in the more moderate conditions of autumn, and particularly in spring. Species richness and diversity indices in the parasitoid community experienced no noticeable change, with values rather similar throughout the year.

In spring, there were a greater number of species competing for a more ephemeral resource; thus, the ability to efficiently detect carrion should be important in this season. Alternatively, in winter, fly species do not strongly compete but instead they should have a relatively better performance under harsher climatic conditions.

Besides the aforementioned fly species, the seasonal variation of taxonomic structure observed in the parasitoid community may also be due to the preferences for different hosts. *Nasonia vitripennis* (Pteromalidae) was the most abundant parasitoid recorded in this study and clearly dominated the assemblages in spring and summer. Its host range is rather broad, including species in the genus *Chrysomya* (Calliphoridae), *Peckia* and *Oxysarcodexia* (Sarcophagidae) and *Musca* (Muscidae) (Carvalho et al. 2003; Marchiori 2004, 2005, 2006), and its predominance coincides with the highest number of blow fly species in the carcasses.

Other species occurring predominantly in spring were the encyrtid *Tachinaephagus*, possibly *T. zealandicus*, which has been previously reported parasitizing fly pupae in Argentina (Oliva 1997) and Brazil (Carvalho et al. 2003). The gregarious nature of *Tachinaephagus*, *N. vitripennis*, and *Aphaereta* sp. may be the reason why these species are so abundant in our catches, reflecting the success of these species in parasitoid community studied here. Gregariousness has been considered a good atribute for the biological control of filth flies, since production costs are substantially lower in social species compared to solitary species of parasitoids (McKay et al. 2007).

Winter and autumn communities of parasitoids were characterized by the occurrence of *Aphaereta* sp. *Alysia alticola* is a Neartic species that was recently reported in Argentina for the first time (Salvo and Battan Horenstein 2008). The results obtained here confirm our hypothesis that the seasonality of regional climate is an important factor structuring communities of blow flies and their associated parasitoids. The pronounced changes displayed in the parasitoid community across the seasons disagree with the observed changes in the community dynamics of leafminers and their parasitoids in central Argentina (Valladares and Salvo 2001). In that study, taxonomic composition of host community showed notorious changes throughout the year, and the parasitoid community displayed little evidence of any seasonal trend in species occurrence. Such discrepancies may be due to the very different nature of the systems involved.

Our study is the first on the community of Diptera and their associated parasitoids for Cordoba. The knowledge of the dynamics of carrion flies and their parasitoids will allow for development of plans for biological control of fly species that have medical and veterinary importance.

**Table 1.**
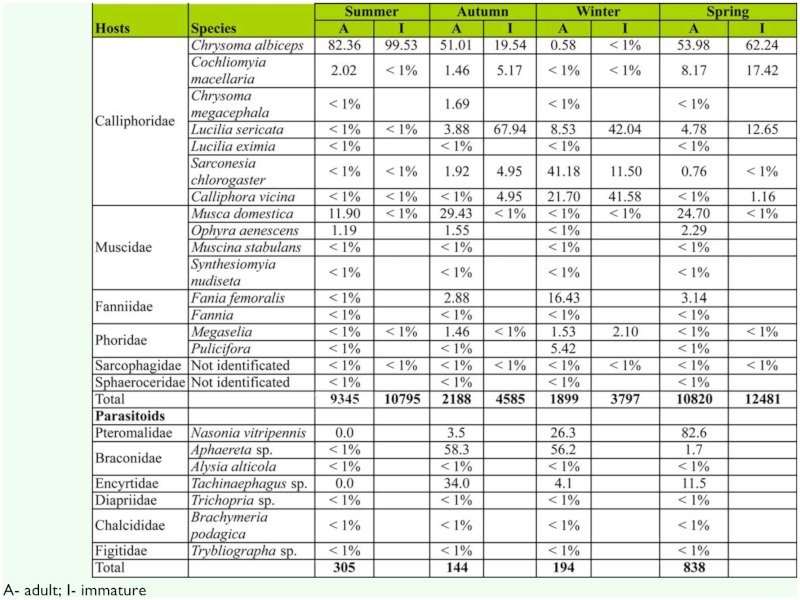
Relative abundance of dipteran (adult and immature stage) and parasitoid (adult stage) communities in the four seasons.

**Table 2.**
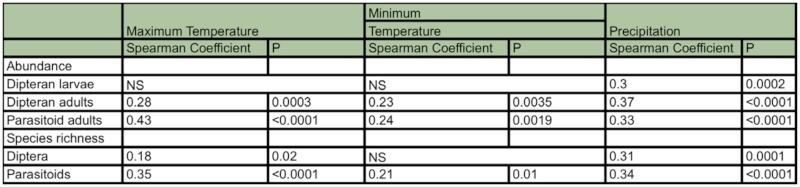
Spearman correlation coefficients between climatic variables and abudance and richness in the dipteran and parasitoid communities. NS indicates no significant correlations among variables.
